# Supplementary material for: Nickel sulfide nanocrystals on nitrogen-doped porous carbon nanotubes with high-efficiency electrocatalysis for room-temperature sodium-sulfur batteries
Source: Nat Commun. 2019 Oct 22;10:4793. doi: 10.1038/s41467-019-11600-3 (PMC6805862; doi:10.1038/s41467-019-11600-3)
Supplement: Supplementary file 1 — Supplementary Information [file 41467_2019_11600_MOESM1_ESM.pdf]

Supplementary Information

NiS_2 nanocrystals implanted nitrogen-doped porous carbon nanotubes with highly-efficient electrocatalytic effect for room-temperature sodium-sulfur batteries

Yan et al.

Supplementary Figures

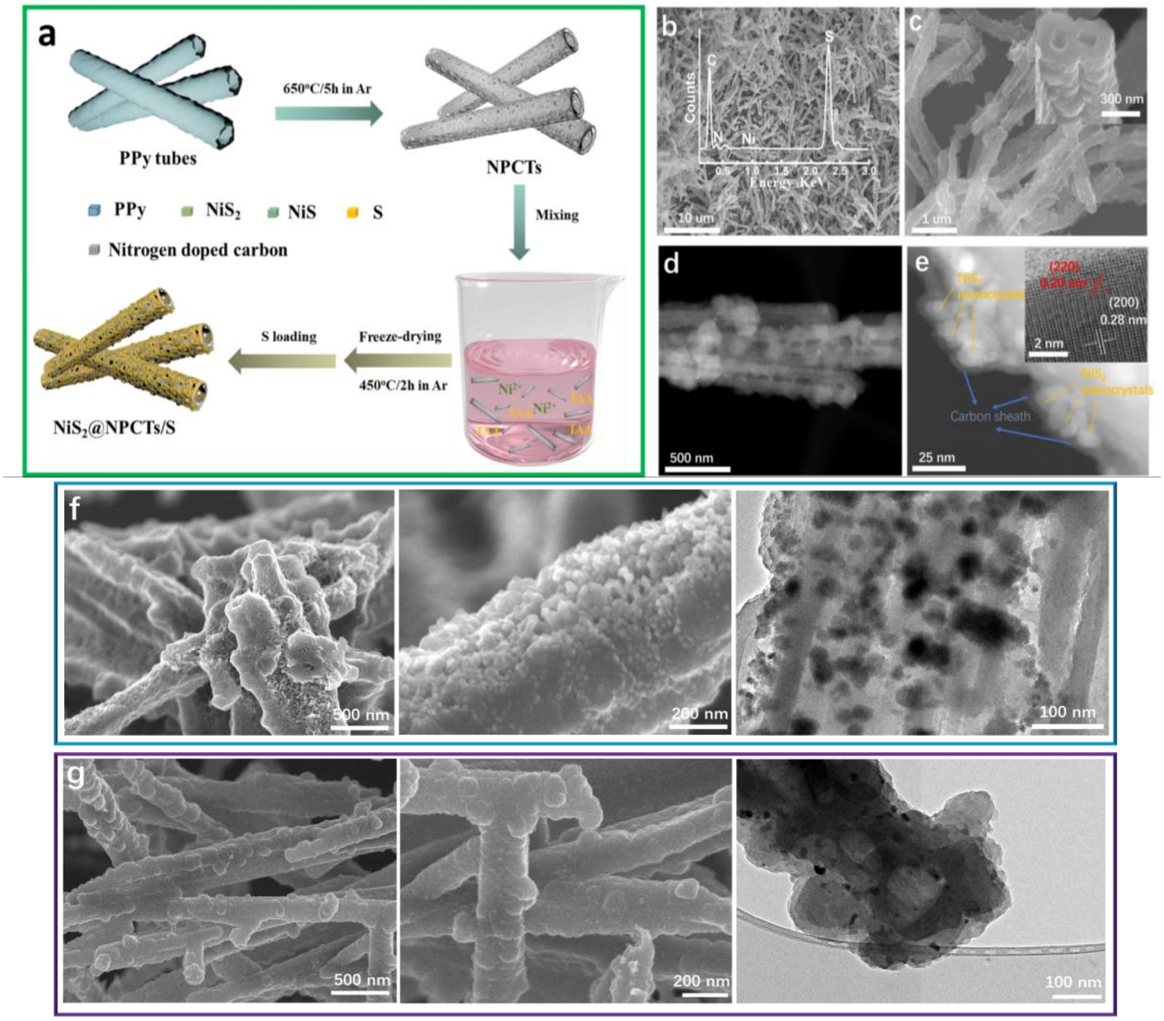

Supplementary Figure 1 | Fabricating procedure, SEM, and HAADF-STEM images of NiS_2@NPCTs/S. **(a)** Schematic of the procedure for fabricating NiS_2@NPCTs/S composite. **(b,c)** SEM images, **(d,e)** High-angle annular dark-field scanning transmission electron microscopy (HAADF-STEM) images. Inset of **(b)** the EDX spectrum of the area. SEM and TEM images of NiS_2@NPCTs/S under different synthesis conditions. **(f)** Stirring without vacuum, **(g)** stirring with vacuum.

The NiS_2@NPCTs/S is prepared by a feasible strategy, which involves freeze-drying method followed by heating treatment of the mixture of NPCTs, nickle salt and thioacetamide (TAA) solution and stirred under vacuum (Supplementary Fig. 1a). The as-prepared NiS_2@NPCTs/S product well maintains the one-dimensional (1D) morphology with an average diameter of ~250 nm (Supplementary Fig. 1b-d). HAADF-STEM image reveals NiS_2 nanocrystals are encapsulated in the NPCTs (Supplementary Fig. 1e). This unique structure has been further illustrated by the enlarged STEM-EDS mapping Supplementary Fig. 2. A comparison experiment to optimize the synthetic procedures has been introduced. As shown in Supplementary Fig. 1f,g, without vacuum treatment, most of the NiS_2 compounds can be visually observed by SEM, indicating the NiS_2 compounds were adsorbed on the exterior of NPCTs. Thus, key point in this synthesis is stirring treatment of the mixture of NPCTs, nickle salt and thioacetamide (TAA) solution under vacuum.

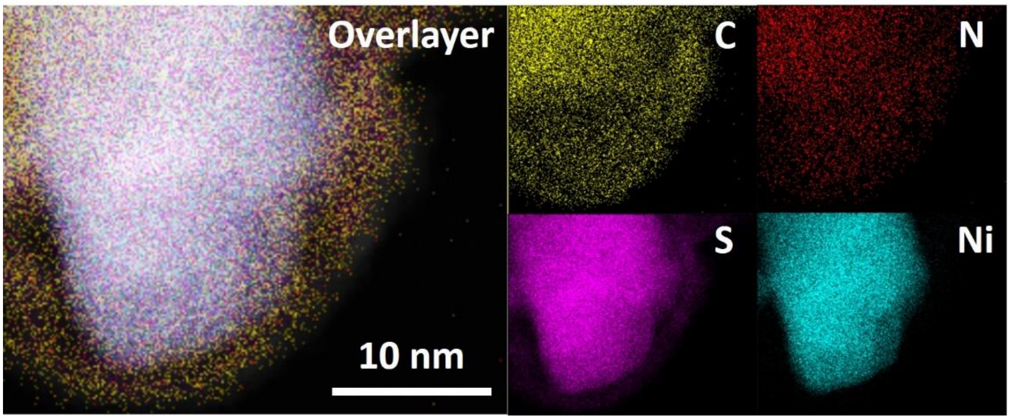

Supplementary Figure 2 | STEM-EDS mapping images of NiS_2@NPCTs/S. Enlarged STEM-EDS mapping images of a single encapsulated NiS_2 nanocrystal of NiS_2@NPCTs/S composite

As illustrated in Supplementary Fig. 2, the multiple cavities of the conductive matrix with relative closed structures are highly contributed to the heating temperature.

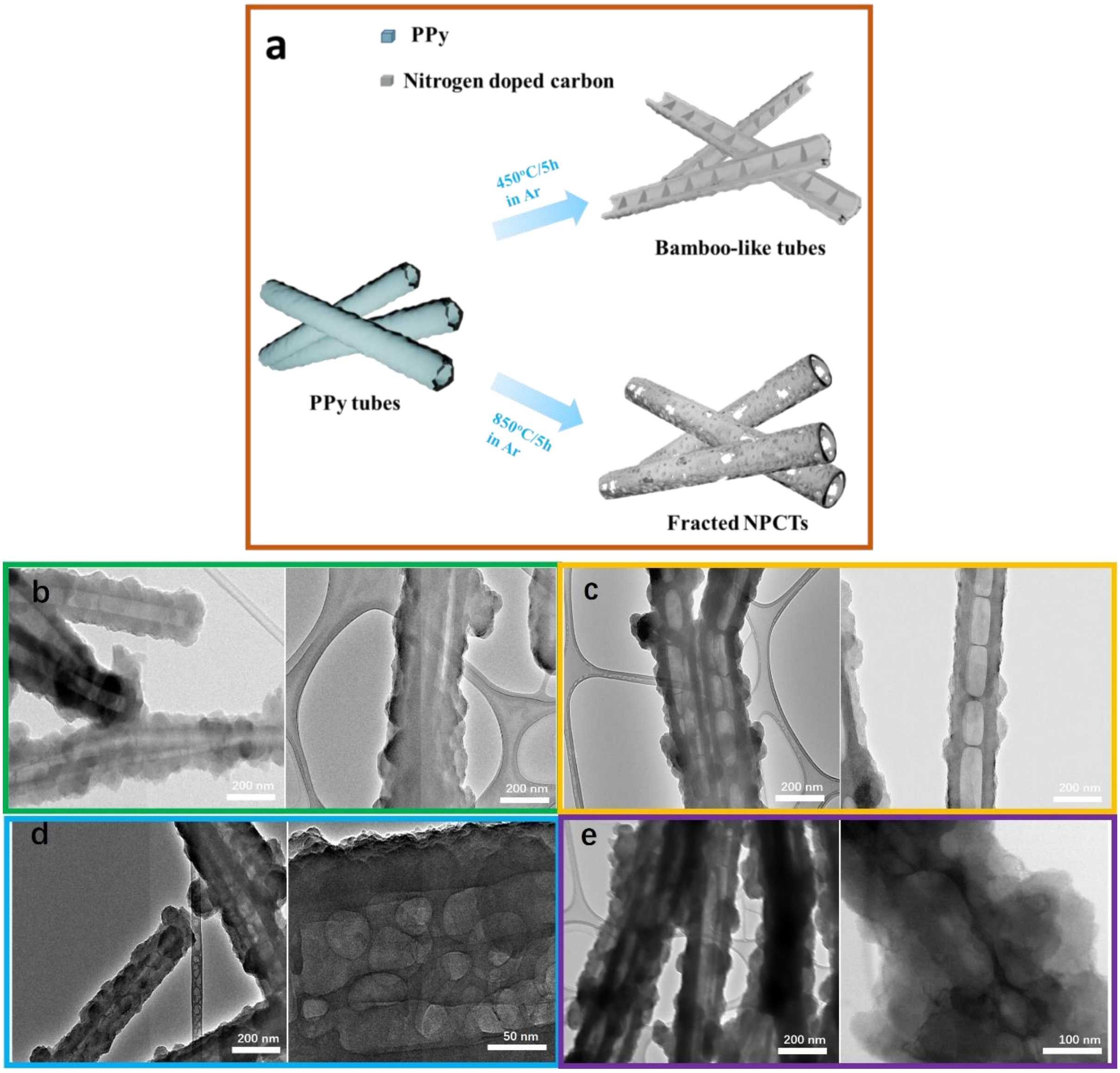

Supplementary Figure 3 | TEM images of N-doped carbon matrixes under different temperatures. **(a)** Schematic of the structure of N-doped carbon matrixes under different temperature. TEM images of **(b)** polypyrrole nanotubes, and its morphology under different carbonization temperature **(c)** 450 °C, **(d)** 650 °C, and **(e)** 850 °C.

Uniform polypyrrole (PPy) nanotubes precursor was prepared by a polymerization method.[1] When carbonized at different temperature, N-doped carbon matrixes with distinguishing fine structures can be formed, respectively. The PPy precursor shrunken and broken at 850 °C and the nanotube morphology of PPy is well kept at 650 °C. The results indicate that NPCTs under 650 °C shows favourable nano-lacunose structure as a sulfur host.

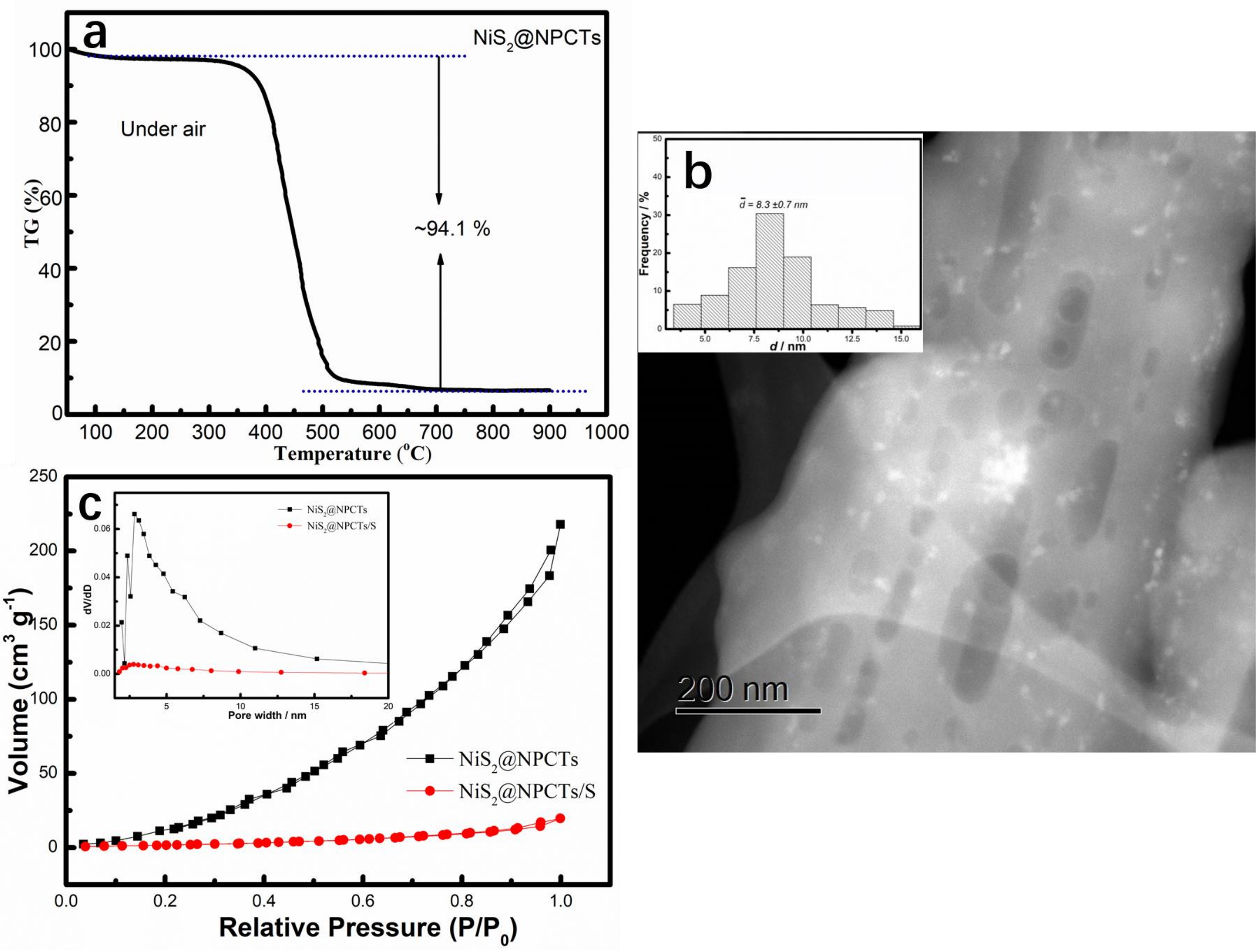

Supplementary Figure 4 | TG and BET of NiS_2@NPCTs matrix and NiS_2@NPCTs/S composite. (a) Thermogravimetry (TG) curve of NiS_2@NPCTs matrix under air flow. **(b)** HAADF-STEM image of NiS_2@NPCTs/S with histogram showing size distribution of NiS_2 nanocrystals based on a count of 150 nanocrystals (inset). **(c)** N_2 absorption/desorption isotherms and pore size distribution (inset) for the pure NiS_2@NPCTs matrix and NiS_2@NPCTs/S composite.

The nitrogen absorption analysis of NiS_2@NPCTs revealed a high specific surface area of 624 m^2 g^{-1} and a hierarchical mesoporous structure. As showed by the pore size distribution, uniform mesopore diameters centred at 2.2–3 nm with total pore volume of 0.44 cm^3 g^{-1} can be observed. After the sulfur infusion, the values of specific surface area and pore volume decrease to 20 m^2 g^{-1} and 0.03 cm^3 g^{-1}, which can be attributed to the distribution of S in both the hollow spaces and the pores of the host. The pore volume variation of 0.41 cm^3 g^{-1} calculated to a maximum of 1.041 g of S embedded in the mesopores for each gram of NiS_2@NPCTs.

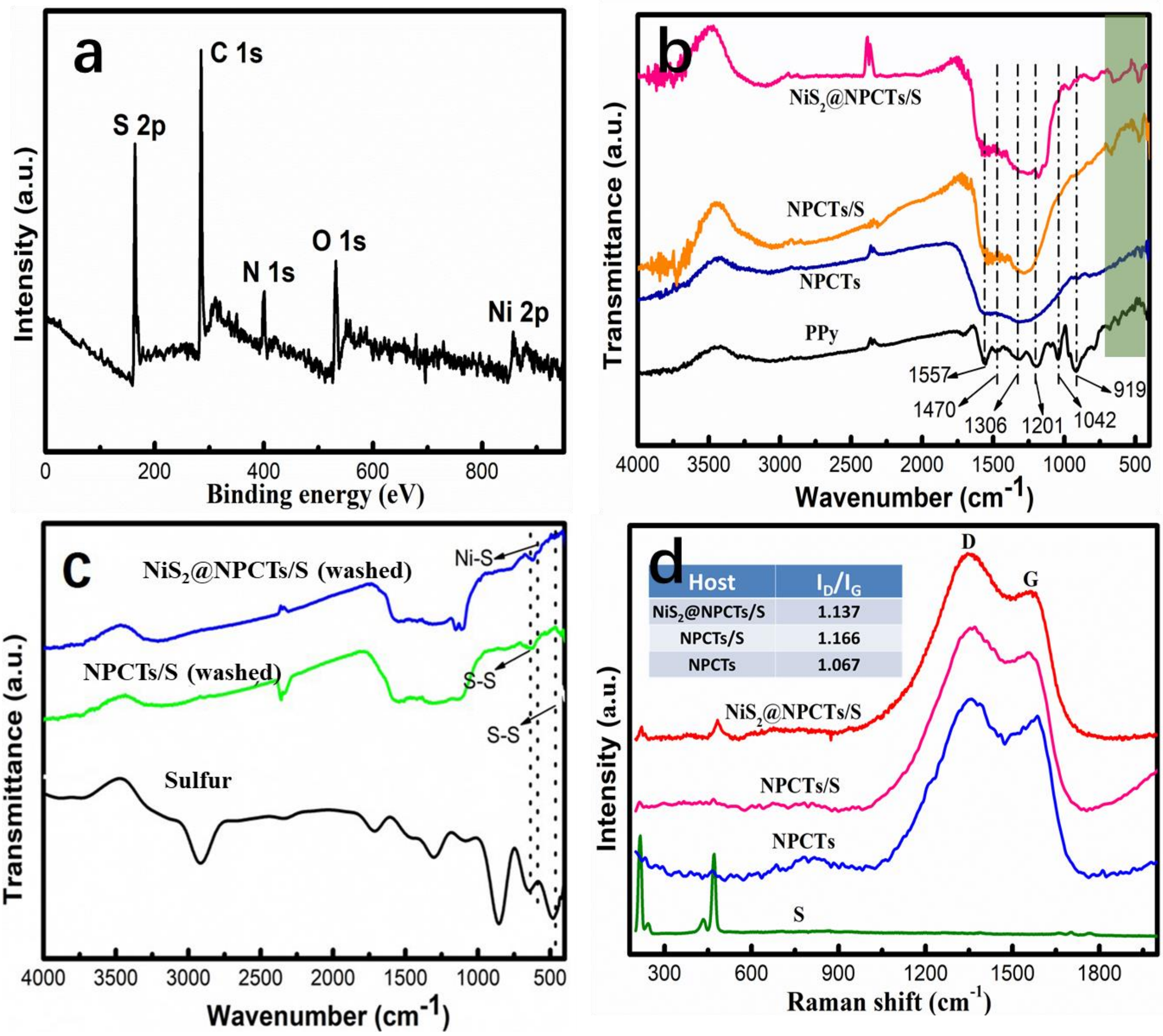

Supplementary Figure 5 | XPS, FT-IR spectra, and Raman spectra analysis. (a) Full XPS spectrum of NiS_2@NPCTs/S composite. **(b)** FT-IR spectra of the PPy, NPCTs, NPCTs/S, and NiS_2@NPCTs/S. **(c)** FT-IR spectra of pure sulfur and carbon disulfide washed NiS_2@NPCTs/S and NPCTs/S composites. **(d)** Raman spectra of the NiS_2@NPCTs/S, NPCTs/S, NPCTs and pure sulfur.

The XPS survey spectrum shows five characteristic peaks at around 164, 285, 401, 531, and 858 eV, corresponding to the S2p, C1s, N1s, O1s, and Ni2p, respectively. The FTIR bands of PPy (Figure S5b) are in good agreement with those reported in the literature.[2] Peaks at 1557 and 1470 cm^{-1} are attributed to the fundamental vibrations of polypyrrole ring, those at 1306 and 1042 cm^{-1} are due to the =C–H in-plane vibrations, the band corresponding to the C–C out of the plane ring deformation vibration is situated at 919 cm^{-1}, and that at 1201 cm^{-1} is due to the C–N stretching vibrations. For NPCTs, however, no obvious characteristic peaks could be assigned to PPy, indicating that PPy has been carbonized to another carbon-based form. Two new peaks located at 458 cm^{-1} and 638 cm^{-1} for both the NPCTs/S and NiS_2@NPCTs/S composites after S impregnation can be attributed to the S-S stretch.[3, 4] Besides, the S-S stretch can still be detected in the CS_2 washed NiS_2@NPCTs/S and NPCTs/S composites, indicating partial S is tightly immobilized in the carbon structure.

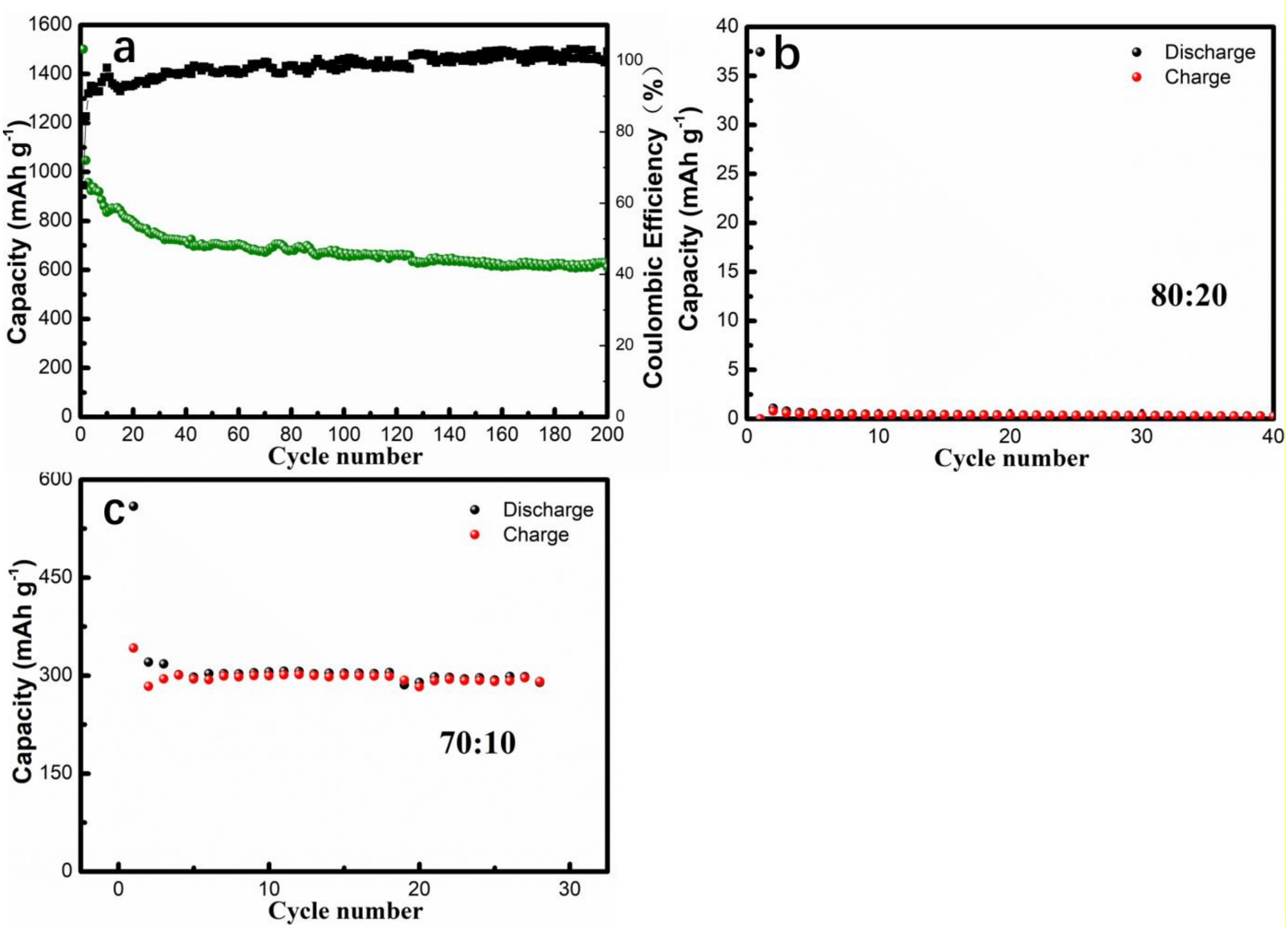

Supplementary Figure 6 | Cycling performance of NiS_2@NPCTs/S composite. **(a)** Cycling performance of NiS_2@NPCTs/S composite at a current density of 0.1 A g^{-1}. The battery performance of the NiS_2@NPCTs/S electrode without carbon black **(b)** S-host and CMC in a weight ratio of 80:20, **(c)** 70:10.

The battery performance without carbon black are shown in Supplementary Fig. 6. Firstly, the working electrodes were fabricated by mixing the S-host and carboxymethyl cellulose (CMC) binder in a weight ratio of 80:20. However, the battery with this ratio has no capacity at all indicating poor connections of the S-host. Considering the electrochemically inactive and nonconductive of binders, we reduced the amount of CMC in this system and tired the ratio of 70:10. The battery with this ratio showed a reversible capacity of 300 mAh g^{-1} at the current density of 0.1 A g^{-1}, which is only half of the performance with carbon black.

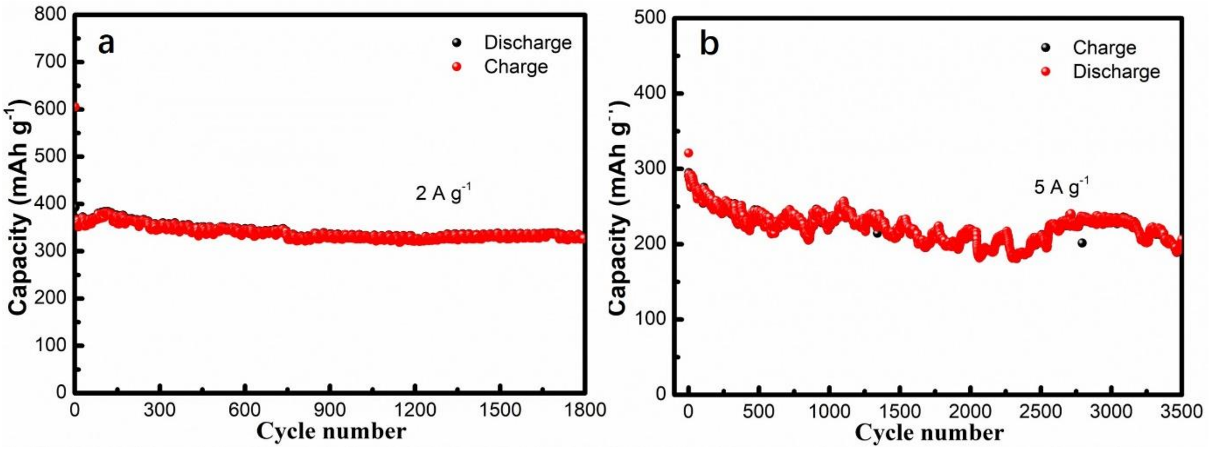

Supplementary Figure 7 | High rate cycling performance. Cycling performance of NiS_2@NPCTs/S at a current density of **(a)** 2 A g^{-1} and **(b)** 5 A g^{-1}.

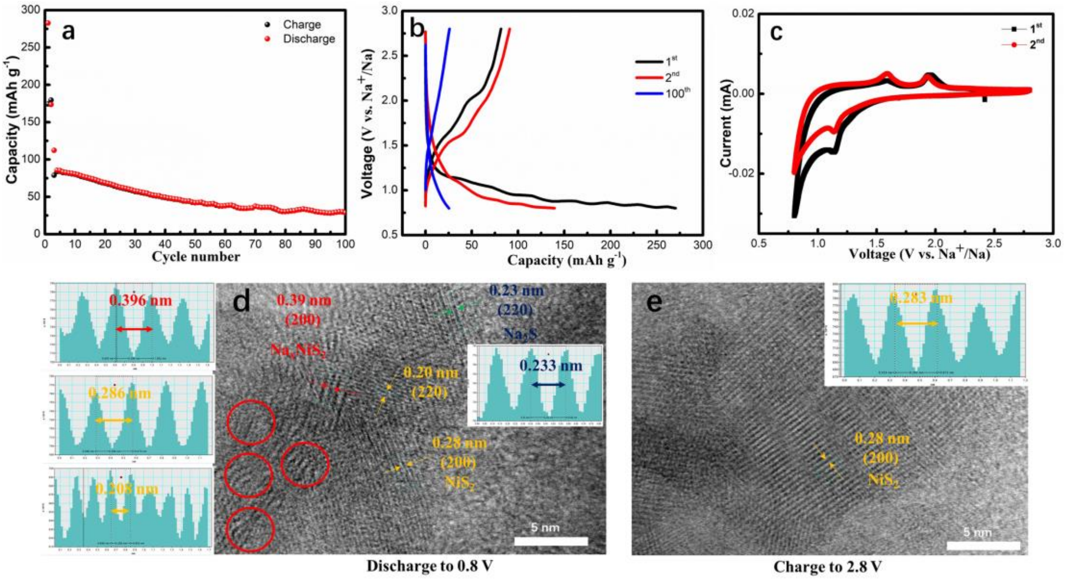

Supplementary Figure 8 | Characterization of mechanism. (a) Cycling performance of NiS_2@NPCTs electrode at a current density of 0.5 A g^{-1}. (b) The corresponding charge/discharge profiles. **(c)** Cyclic voltammogram of NiS_2@NPCTs with the scan rate of 0.1 mV/s. Ex-situ HRTEM images of the NiS_2@NPCTs/S electrode **(d)** Discharge to 0.8 V, **(e)** Charge to 2.8 V.

The discharge curve of NiS_2@NPCTs shows one plateau (1.1 V) and a long tail around 0.8 V in discharge, which is well accordant with its Cyclic voltammogram. The cathodic peak at 1.1 V can be attributed to the reaction of $NiS_2 + xNa+ + xe- \rightarrow NaxNiS_2$. The long tail around 0.8 V can be attributed to further Na+ intercalation and formation of solid electrolyte interphase (SEI) film. Besides, we found the capacity retention of NiS_2@NPCTs is very low between 0.8-2.8 V, and only 25 mAh/g remained after 100 cycles. Thus, the capacity contribution of NiS_2 to this system is negligible when compared to the NiS_2@NPCTs/S (more than 600 mAh/g for 200 cycles at 0.1 A/g). We further disclosed the mechanism by ex-situ HRTEM (high resolution transmission electron microscopy) of the NiS_2@NPCTs/S electrode during the sodiation–desodiation of Na ions. When discharged to 0.8 V, Supplementary Fig. 8d demonstrates the intercalation reaction of NiS_2 with the lattice spacing of (200) increases to 0.39 nm. Planes of NiS_2 are still discovered, indicating that the intercalation reaction is incomplete. The Na_2S as the final discharge product of sulfur can also be found. The detected NiS_2 and Na_2S at 0.8 V with low crystallinity, which agrees well with the in-situ synchrotron results. When fully charged to 2.8 V, the lattice fringe of 0.28 nm is assigned to the (200) plane of NiS_2. According to the comparison in electrochemical behavior, in-situ synchrotron, and ex-situ HRTEM,the overlapped discharge plateau in NiS_2@NPCTs/S electrode can not only be attributed to the reaction between Na+ and S, but also partial NiS_2 react with sodium ion based on the mechanism: $NiS_2 + xNa+ + xe- \rightarrow NaxNiS_2$.

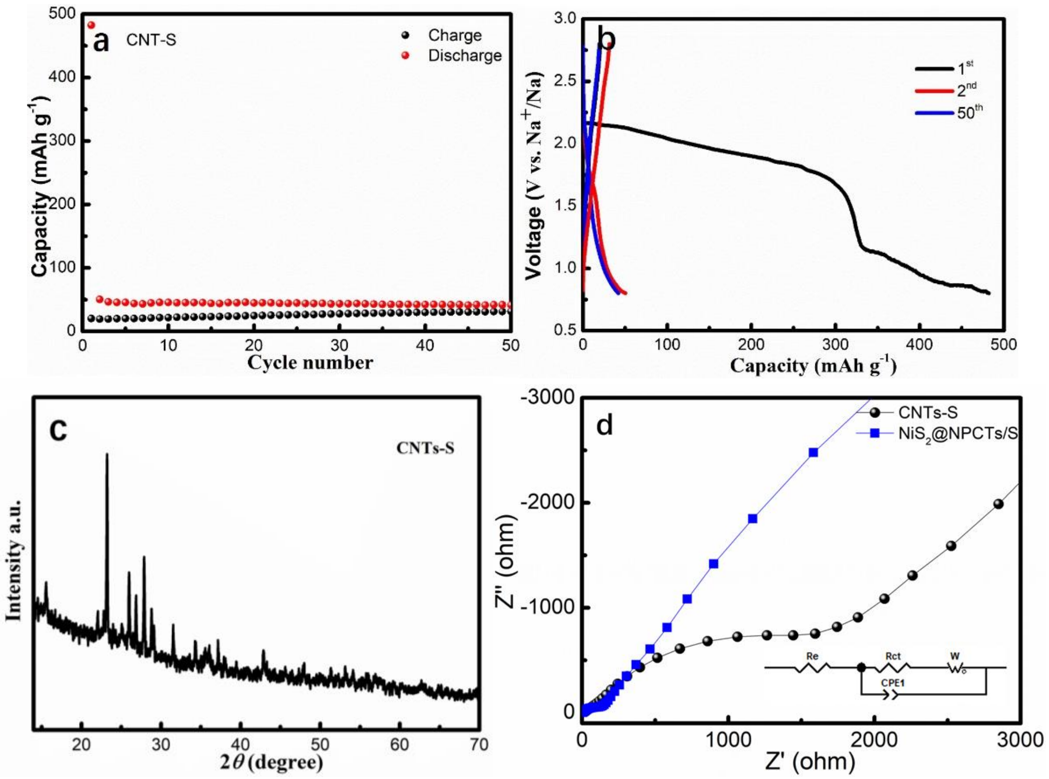

Supplementary Figure 9 | Characterization of CNTs-S. **(a)** Cycling performance of commercial carbon nanotubes (CNTs-S) at a current density of 0.5 A g^{-1}. **(b)** The corresponding charge/discharge profiles. **(c)** XRD pattern. **(d)** Nyquist plots for CNTs-S and NiS_2@NPCTs/S composite after 10 cycles.

Supplementary Figure 9b shows a longer plateau at 2.2 V than that of NiS_2@NPCTs/S electrode. To understand the reason for this phenomenon, A comparison XRD pattern of these two samples are discussed. The XRD pattern in Supplementary Figure 9c reveals a much stronger crystalline state of sulfur in CNTs-S, indicating the poor encapsulation in CNTs. However, sulfur encapsulated in NiS_2@NPCTs/S shows much reduced intensity of XRD peaks compared with that of pure elemental sulfur (manuscript Figure 1d). As we mentioned in our manuscript, the plateau around 2.2 V is highly related to the reduction of S_8 to form long-chain polysulfides. Thus, CNTs-S electrode with more crystalline S_8 shows a stronger reduction plateau of S_8 at 2.2 V.

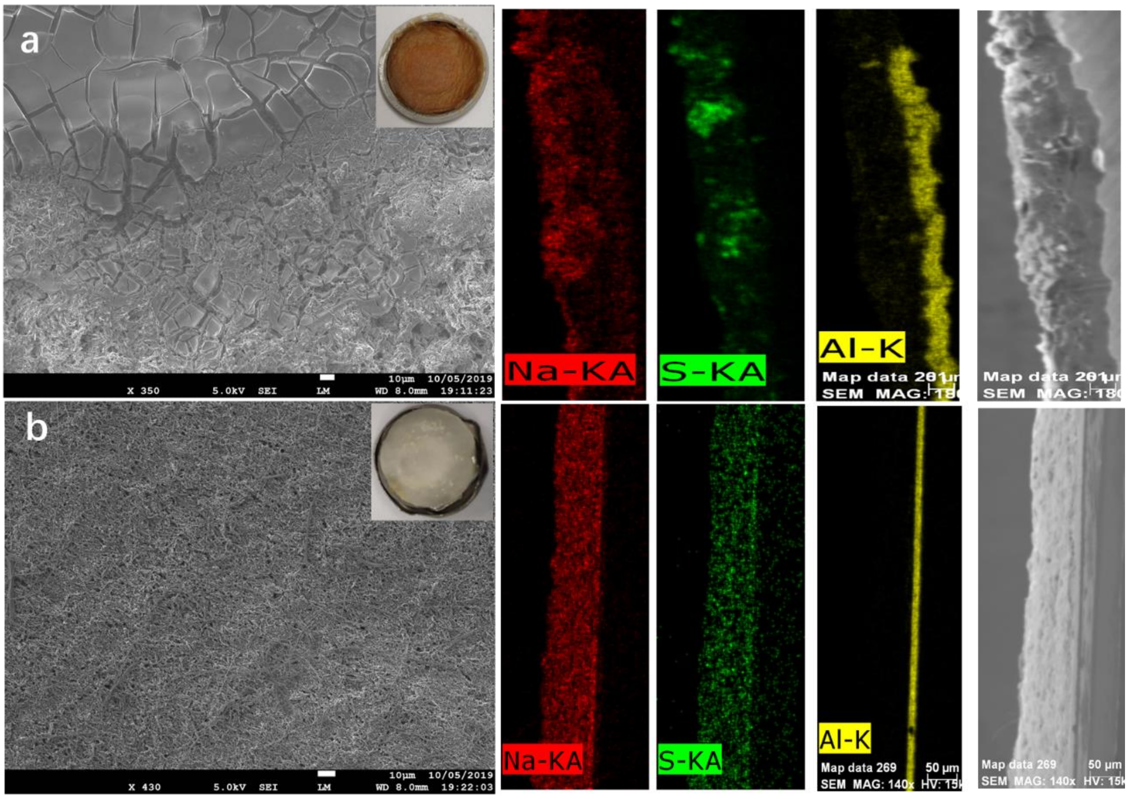

Supplementary Figure 10 | SEM, cross-profile EDS mapping images. (a) CNTs-S and **(b)** NiS_2@NPCTs/S electrodes with digital photographs of the corresponding separator.

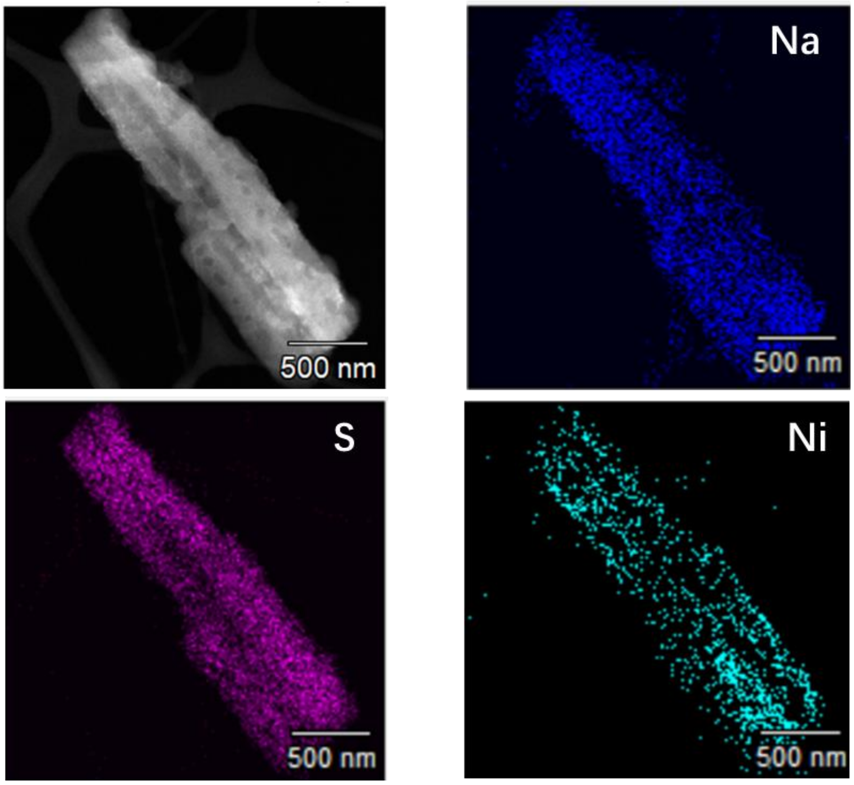

Supplementary Figure 11 | STEM-EDS mapping images of the NiS_2@NPCTs/S electrode under sodiated state.

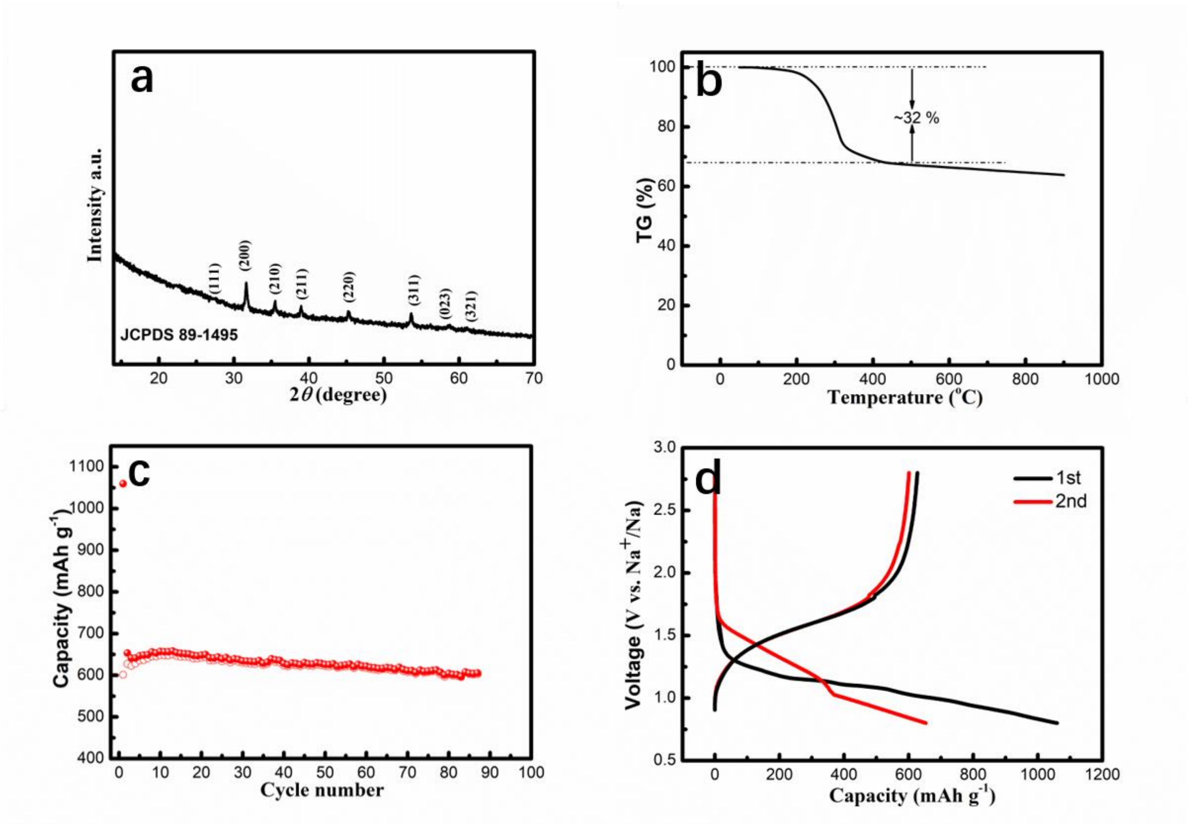

Supplementary Figure 12 | Characterization of NiS_2@NPCTs/S32. (a) XRD patterns, **(b)** Thermogravimetry curve, **(c)** Cycling performance at a current density of 1 A g^{-1}, **(d)** the corresponding charge/discharge profiles.

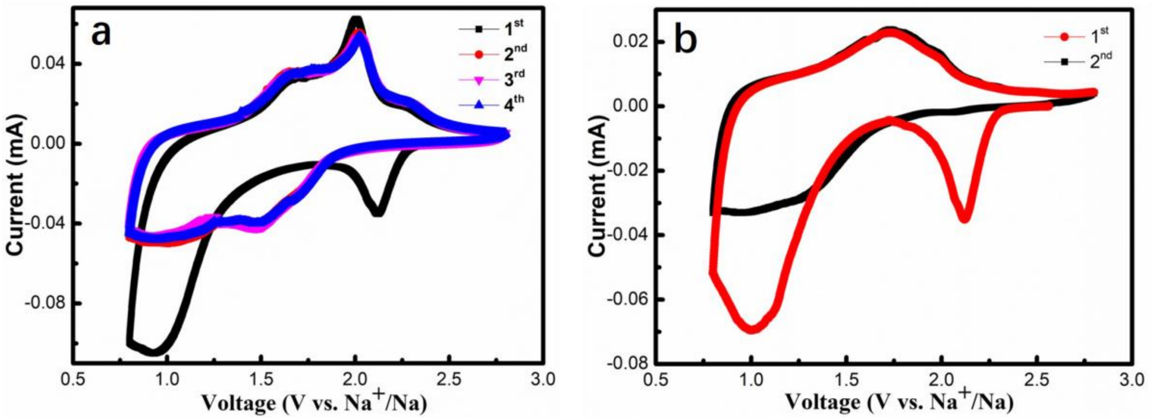

Supplementary Figure 13 | Electrochemical characterization. (a) Cyclic voltammogram of NiS$_2$@NPCTs/S composite in the first four cycles. **(b)** Cyclic voltammogram of NPCTs/S cathode for the first two cycles.

Supplementary Fig. 11a presents the CV curves of the RT-Na/NiS$_2$@NPCTs/S cell at a scan rate of 0.1 mV s^{-1} between 0.8-2.8 V. There are two prominent peaks centred at 2.12 and 0.85 V during the first cathodic scan. The peak around 2.12 V can be attributed to the solid-liquid transition from elemental sulfur to long-chain polysulfides, and the soluble long-chain polysulfides are gradually transferred to less soluble short-chain Na_2S_4 in the following reactions. The sharp peak at 0.85 V likely corresponds to the formation of Na_2S during the further sodiation.[5, 6, 7] In the following cathodic scan, two major reduction peaks at approximately 1.51 and 0.95 V and a small shoulder at approximately 1.68 V are highly repeatable without current attenuation, indicating reversible reaction mechanism with high capacity retention in this system. The peaks in the region of 1.68-1.51 V can be ascribed to a liquid–liquid reaction between the dissolved Na_2S_6 to Na_2S_4, and the other one located at 0.95 V is supposed to the formation of short-chain Na_2S_2 and Na_2S.[8, 9, 10] Three corresponding oxidation peaks (two major and one minor peaks) also appear in the charge process, suggesting that the redox process of NiS$_2$@NPCTs/S electrode is highly reversible. The three repeatable oxidation peaks at 1.65, 2.01, and 2.28 V could be ascribed to the staged oxidation process of Na_2S to short chain Na_2S_4, and then to long-chain sodium polysulfides.[11] Similar redox behaviors were also observed for NPCTs/S electrode. In contrast with the NPCTs/S, the CV of the NiS$_2$@NPCTs/S electrode shows prominent cathodic/anodic peaks with much higher intensity. Thus, we speculate that the implanted NiS$_2$ nanocrystals not only constrain polysulfide disslution but also facilitate reaction kinetics towards high sulfur utilization and high capacity.

Supplementary Tables

Supplementary Table 1| Results from previous room-termperautre sodium sulfur batteries cathode

Active material	Loading sulfur	Electrolyte	Current collector	Working current	Cycle number	Retained discharge capacity	Ref
S@C	35%	1M $NaPF_6$ with 0.25M $NaNO_3$ in TEGDME	Stainless steel discs	1C	1500	300mAh g^{-1}	5
NGNS/S	25%	1M NaClO4 in EC:DMC:PC	Al foil	0.1C	300	66mAh g^{-1}	6
S@iMCHS	59%	1M NaClO4 in EC:PC with 5%FEC	Al foil	100mA g^{-1}	200	292mAh g^{-1}	28
CSB@TiO_2	60%	1M NaClO4 in EC:DEC	Free-standing	1A g^{-1}<br>2A g^{-1}	1400<br>3000	524mAh g^{-1}<br>382mAh g^{-1}	30
ZIF-8/S	50%	1M NaClO4 in TEGDME	Al foil	0.2C	250	500mAh g^{-1}	31
c-PANS	31%	0.8M NaClO4 in EC:DEC	Al foil	220mA g^{-1}	500	180mAh g^{-1}	32
S/(CHNBs@PCNFs)	70%	1M NaClO4 in EC:PC with 5%FEC	Al foil	2C	400	256mAh g^{-1}	33
MCPS1	47%	1M NaClO4 in EC:PC with 5%FEC	Carbon coated Al foil	0.1C	50	800mAh g^{-1}	35
S@Con-HC	47%	1M NaClO4 in EC:PC with 5%FEC	Cu foil	100mA g^{-1}	600	508mAh g^{-1}	36
NiS_2@NPCTs/S	56%	1M NaClO4 in EC:PC with 5%FEC	Al foil	0.1A g^{-1}<br>1A g^{-1}<br>2A g^{-1}<br>5A g^{-1}	200<br>1400<br>1800<br>3500	650mAh g^{-1}<br>401mAh g^{-1}<br>327mAh g^{-1}<br>208mAh g^{-1}	**This work**

Supplementary Table 2| Calculated binding energies (unit in eV) for sodium polysulfide on NiS_2 nanocrystal (100), N-doped carbon, and carbon, respectively.

sodium polysulfide	NiS_2 nanocrystal (100)	N-doped carbon	carbon
Na_2S_6	0.79	0.57	0.09
Na_2S_4	0.7	0.50	0.16
Na_2S_2	1.6	0.71	0.57
Na_2S	2.4	0.65	0.41

Supplementary References

[1] H. Guo, B. Ruan, L. Liu, L. Zhang, Z. Tao, S. Chou, J. Wang, H. Liu, *Small* **2017**, 13, 1700920.
[2] J. Zheng, W. Zhou, Y. Ma, W. Cao, C. Wang, L. Guo, *Chem. Commun.* **2015**, 51, 12863.
[3] Y. Xu, C. Zhang, M. Zhou, Q. Fu, C. Zhao, M. Wu, Y. Lei, *Nat. Commun.* **2018**, 9, 1720.
[4] W. Li, M. Zhou, H. Li, K. Wang, S. Cheng, K. Jiang, *Energy Environ. Sci.* **2015**, 8, 2916.
[5] K. J. Zhu, G. Liu, Y. J. Wang, J. Liu, S. T. Li, L. Y. Yang, S. L. Liu, H. Wang, T. Xie, *Materials Letters* **2017**, 197, 180-183.
[6] a) X. Yu, J. Xie, Y. Li, H. Huang, C. Lai, K. Wang, *J. Power Sources* **2005**, 146, 335; b) S. Wei, L. Ma, K. E. Hendrickson, Z. Tu, L. A. Archer, *J. Am. Chem. Soc.* **2015**, 137, 12143.
[7] Y. X. Wang, J. Yang, W. Lai, S. L. Chou, Q. F. Gu, H. K. Liu, D. Zhao, S. X. Dou, *J. Am. Chem. Soc.* **2016**, 138, 16576.
[8] X. Yu, A. Manthiram, *J. Phys. Chem. Lett.* **2014**, 5, 1943.
[9] a) D. Ma, Y. Li, J. Yang, H. Mi, S. Luo, L. Deng, C. Yan, M. Rauf, P. Zhang, X. Sun, X. Ren, J. Li, H. Zhang, *Adv. Funct. Mater.* **2018**, 28, 1705537; b) X. Yu, A. Manthiram, *ChemElectroChem* **2014**, 1, 1275.
[10] S. Wenzel, H. Metelmann, C. Raiß, A. K. Dürr, J. Janek, P. Adelhelm, *J. Power Sources* **2013**, 243, 758.
[11] B. W. Zhang, Y. D. Liu, Y. X. Wang, L. Zhang, M. Z. Chen, W. H. Lai, S. L. Chou, H. K. Liu, S. X. Dou, *ACS Appl. Mater. Interfaces* **2017**, 9, 24446.
